# Contribution of QuantiFERON-TB Gold-in-Tube to the Diagnosis of *Mycobacterium tuberculosis* Infection in Young Children in a Low TB Prevalence Country

**DOI:** 10.3389/fped.2019.00291

**Published:** 2019-07-18

**Authors:** Sara Debulpaep, Véronique Corbière, Jack Levy, Petra Schelstraete, Koen Vanden Driessche, Françoise Mascart, Françoise Mouchet

**Affiliations:** ^1^Pediatric Department, CHU Saint Pierre University Hospital, Université Libre de Bruxelles, Brussels, Belgium; ^2^Laboratory of Vaccinology and Mucosal Immunity, Université Libre de Bruxelles, Brussels, Belgium; ^3^Pediatric Department, Ghent University Hospital, Ghent, Belgium; ^4^Division of Pediatric Pulmonology and Infectious Diseases, Pediatric Department, Ghent University Hospital, Ghent, Belgium; ^5^Division of Pediatric Pulmonology, Pediatric Department, University Hospital Antwerp, Antwerp, Belgium; ^6^Department of Laboratory Medicine, Radboud University Medical Center, Institute for Molecular Life Sciences, Nijmegen, Netherlands; ^7^Immunobiology Clinic, Hôpital Erasme, Université Libre de Bruxelles, Brussels, Belgium

**Keywords:** tuberculosis, latent tuberculosis infection, tuberculin skin test, interferon gamma release assay, QuantiFERON, children, contact screening

## Abstract

**Introduction:** Interferon Gamma Release Assay (IGRA) has proven to be a useful test to evaluate the immune response to *Mycobacterium tuberculosis* antigens in children over the age of 5 years as an alternative to tuberculin skin testing (TST). Much less is known about its performance in younger children, who are at higher risk for developing tuberculosis (TB) disease after exposure. We aimed to evaluate the accuracy of using IGRA in TB screening in this population.

**Methods:** Children below the age of 5 years at high risk for TB infection were prospectively enrolled, to compare the performance of TST and the QuantiFERON-TB Gold-In-Tube test (QFT). Children were treated in accordance with the diagnosis made at baseline and followed-up for 12 months.

**Results:** We included a total of 60 children of which 97 blood samples were available for analysis. There was 90.72% agreement between TST and QFT (Kappa test 0.59, moderate agreement). With TST as a reference, the QFT positive predictive value was 0.72 and the negative predictive value 0.93. Discordant results were observed with 6% TST+/QFT– paired tests. When we restricted the comparison of TST and QFT to non-BCG-vaccinated children, the degree of agreement was more substantial (95%, Kappa test 0.75) and the negative predictive value was 0.99. We observed 3% discordant TST–/QFT+ results. All children with active TB disease had concordant positive QFT results, with QFT values above 4.00 IU/ml.

**Conclusion:** In a low TB prevalence country, serial testing of QFT was found to produce a moderate agreement with TST results. False positive QFT results would have been eliminated by using a higher cutoff without misdiagnosing the children with TB disease. Some of the false negative QFT results could be explained by false positive TST results on consecutive testing. For now the most prudent approach would be to consider discordant QFT–/TST+ results as false negative QFT results, taking into account the young age of our population and the potential risk for evolution to active TB disease.

## Introduction

According to the World Health Organization (WHO), one million children fell ill with tuberculosis (TB) in 2017. A higher percentage (15%) of children died from the disease compared to their share of estimated cases, suggesting their reduced access to diagnosis and treatment (1).

Following infection, young children progress more frequently and more rapidly to active TB disease compared to older children and adults (2–5). Treatment of latent tuberculous infection (LTBI) reduces the risk of disease progression, which is especially crucial for young children (6, 7). The WHO therefore recommends to screen young children (<5 years old) who have recently been in contact with a person affected by pulmonary TB and to initiate LTBI treatment even before infection can be demonstrated. Tuberculin skin testing (TST) is the recommended method for evaluating young children for TB infection (1, 7, 8). Difficulties, however, hamper the use of the TST with result interpretation, the need for a follow-up visit by the patient and false-positive results caused by cross-reaction with the Bacillus Calmette-Guérin (BCG) vaccine and with non-tuberculous mycobacteria (NTM) (9). Another diagnostic test, the Interferon Gamma Release Assay (IGRA), is available. IGRA is an *in vitro* blood test, detecting the release of interferon-gamma (IFN-γ) by circulating T cells following stimulation with antigens unique to *Mycobacterium (M.) tuberculosis* and three NTM: *M. kansasii, M. szulgai*, and *M. marinum* (10). IGRA results are unaffected by previous BCG vaccination.

Guidelines for the use of TST and/or IGRA as a screening tool for young children are heterogeneous (11–13). There is currently no consensus about the screening policy for identifying children at risk of TB infection. The United Kingdom National Institute for Health and Care Excellence (NICE) (14) and the Centers for Disease Control (CDC) (7) recommend screening children younger than 5 years for TB using TST only. The guidance from the European Center for Disease Prevention and Control (ECDC) states that IGRA testing should not replace TST after exposure to an infectious TB case but may be used, in addition to TST, as part of an overall risk assessment. Any positive result should be considered. For children with low risk, without exposure, IGRA can be used to rule out false positive TST reactions caused by BCG vaccination or after exposure to NTM with a two-step approach (8, 15). This study aims to evaluate the accuracy of IGRA for the screening of TB infection in children aged <5 years in Belgium, a low TB prevalence country with <10 cases per 100,000 population (1).

## Patients and Methods

Children aged 0–5 years assessed for TB between September 2008 and July 2011 at the CHU Saint Pierre, a university-affiliated hospital located in Brussels, were prospectively included in this study. TB screening was performed either in the context of contact with contagious cases in household settings, schools or day-care centers or as part of immigration screening or investigations for suspected active TB disease with clinical or radiological relevant signs.

Clinicians recorded prospectively demographic features, history of exposure to an active TB disease case, clinical history and BCG vaccination status (confirmed by reviewing the immunization record, if available, or by the presence of a scar). Human immunodeficiency virus serology results were recorded, if available, but testing was not performed as a part of this study.

Trained nurses performed TST using the Mantoux method with tuberculin-purified protein derivative RT23 (2 tuberculin units; Staten Serum Institute) and interpreted the result after 48–72 h by measuring the transversal diameter of induration in millimeters (mm). A TST result ≥ 5 mm was considered positive for children with recent contact with a TB case and a TST result of ≥10 mm for the other children, according to national guidelines (Table 1) (16, 17).

**Table 1:** (Addendum): General interpretation criteria of the tuberculin test^*^.

**Diameter of the induration**	**Interpretation**
<5 mm	**Negative**
5–9 mm	**Negative**
	**Positive** - In case of infection with HIV or severe immune deficiency - In young children <5 years of age, who have recently been in contact with an infectious patient or who are immune-deprived or who show a clinical manifestation of active tuberculosis
	**Doubtful** - In persons ≥5 years of age: in case of close contact with an infectious tuberculosis patient - In children <5 years of age or in persons ≥65 years of age: in the absence of risk factors
10–17 mm	**Positive** - In case of close contact with an infectious tuberculosis patient - And/or when there is an increased risk of infection or tuberculosis - In all children, regardless of their age.
	**Doubtful** - In the absence of a risk factor - And/or in the case of BCG
≥18 mm	**Positive**

^*^Translation from “Recommendations regarding the prevention of tuberculosis in healthcare facilities” of the Belgian Superior Health Council. 2013 Report N umber: HGR 8579 (16).

* *This table is under revision and new guidelines will be published in 2019*.

QuantiFERON-TB Gold-In-Tube (QFT), a third-generation IGRA test, evaluates the *in vitro* release of IFN-γ in three different blood collection tubes. The first tube is coated with TB-specific Antigen: ESAT-6, CFP-10, and TB7.7. The second tube is saline, used as a negative control (Nil), that gives information about background IFN-γ secretion (IU/ml). The third tube is a positive control (PHA mitogen) that evaluates the global ability to produce IFN-γ. According to manufacturer's interpretation criteria, a QFT value (IU/ml) is considered positive, for an IFN-γ response to TB Antigen, if significantly above the Nil value (TB Antigen minus Nil) using the recommended threshold ≥0.35 IU/ml and ≥25% of the Nil value. A test is indeterminate if the Nil value is >8.0 IU/ml or if PHA mitogen value minus Nil value is <0.5 IU/ml. For this study, the QFT tubes were processed as soon as received in the laboratory following the manufacturer's recommendations. Plasma samples were stored at −20°C after incubation for at least 16 h and assays were performed once a month.

In the absence of a gold standard to diagnose LTBI, TST was used as the surrogate reference. When TST was positive or in the presence of clinical signs, a chest X-ray was performed.

In case of any radiological or clinical suspicion of TB disease, further investigations were pursued, including microscopy (auramine staining), molecular testing by polymerase chain reaction (PCR) and mycobacterial cultures on three consecutive early-morning gastric aspirate samples in case of suspicion of a pulmonary TB disease or on other samples accordingly to the extra-pulmonary site involved.

Children with a negative TST and without any signs of active TB disease, exposed during the previous 8 weeks to an infectious TB patient, underwent repeated screening, including a TST and a QFT, at least 8 weeks after the latest exposure. During this interval, children received window period prophylactic treatment. We considered children not infected by *M. tuberculosis* if they had a negative TST result more than 8 weeks after the last exposure and if clinical examination provided no argument for illness.

LTBI was diagnosed in children who had a positive TST, normal chest X-ray and who remained asymptomatic during the 12-month follow-up period. Active TB disease was confirmed in case of bacteriologically proven disease and diagnosed as highly probable in children with TB suggestive clinical symptoms and/or radiographic findings, and TST positive result with a response to treatment included in the follow-up. Additional QFT, during follow-up, were performed whenever venous puncture was considered necessary for clinical purposes (evaluation of inflammatory status and/or monitoring of adverse drug reactions) in children with LTBI or TB disease.

After a 12-month follow-up, based on TST results, symptoms, investigations and evolution, all children received a final diagnosis. We compared the results of QFT and TST only if both tests were performed within a 2-week interval. If the initial TST result was more than 10 mm, it was not repeated, and QFT controls were compared to the first positive TST.

Data were analyzed using the GraphPad Prism Software version 7.0b for Windows (GraphPad Software, San Diego, CA, USA, www.graphpad.com). TST and QFT agreements were calculated by the kappa-statistic, a measure of inter-rater agreement between both ratings. The kappa-statistic measure of agreement was scaled to be 0 when the amount of agreement was what would be expected to be observed by chance and 1 if there was perfect agreement. Indeterminate QFT results were excluded from the analysis of concordance with TST. A non-parametric Spearman test analyzed correlations between the TST and QFT values.

## Ethical Approval

Oral and/or written consent was obtained from parents or legal guardians and registered.

## Results

We enrolled 66 children in the study. Out of these, six children were excluded from the final analysis: one child had a confirmed *M. avium* infection, one child's QFT test was lost during transport, one child's QFT test was not done simultaneously with the TST, and three children were lost during follow-up (Table 2). The median age of the included children was 19.5 months (range: 14 days−59 months), with 35/60 children (58%) being younger than 2 years. The study population included 34/60 males (57%), and a minority of the enrolled children (13/60, 22%) were foreign-born. None of the children received immunosuppressive therapy, had a history of LTBI or active TB disease or a chronic medical condition. BCG vaccination was documented for 11/60 children (18%).

**Table 2:** Characteristics of the study population (*n* = 60 children).

**Median age years (age range)**	**19.5 months (14 days−59 months)**
Male	34/60 (57%)
Age <2 years	35/60 (58%)
Foreign born	13/60 (22%)
No BCG vaccination	49/60 (83%)
Exposure to tuberculosis	
Group 1: Known exposure	51/60(85%)
Group 2: High tuberculosis prevalence country	6/60 (10%)
Group 3: Clinical suspicion (no known contact exposure)	3/60(5%)

*BCG, Bacillus Calmette Guerin*.

**Figure 1:**
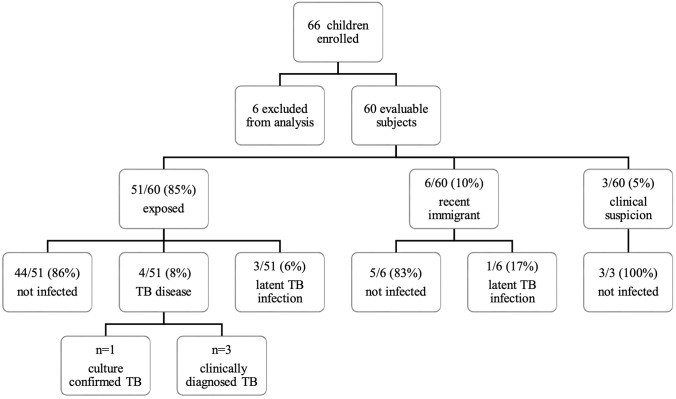
Flowchart of inclusion and classification. Sixty-six children were enrolled in the study. Six children were excluded from analysis: one child had a confirmed *Mycobacterium avium* infection, one child's QFT sample was lost during transport, one child's QFT was not done simultaneously with the TST, and three children were lost during follow-up. Among the 60 remaining children, tests were requested in 51 children after exposition to a contagious TB case, in six due to recent immigration and in three based on a clinical suspicion of TB disease (no known contact exposure). QFT, QuantiFERON®-TB gold in-tube test; TB, tuberculosis; TST, tuberculin skin test.

Among the 60 children included in the final analysis, tests were requested for 51 children due to exposure to a contagious TB case, for six due to recent immigration from a high TB prevalence country and for three due to clinical suspicion of active TB disease (but no known contact exposure) (Figure 1).

At the end of the 12-month follow-up period, among the subgroup of exposed children (*n* = 51), we classified 44 (86%) of them as non-infected, four (8%) as having an active TB disease and three (6%) as being LTBI.

Reference patient numbers are listed in Table 3. Among the seven children classified as *M. tuberculosis* infected (LTBI and active TB disease combined), the first performed TST was ≥10 mm for all but one child, whose diagnosis was confirmed as active TB disease (patient no. 60 in Table 3). He demonstrated a negative TST (0 mm) at inclusion but turned positive (12 mm) at the second evaluation. Among recently immigrated children, we classified 5/6 (83%) as non-infected and 1/6 (17%) as LTBI. None of the children screened for suspected clinical TB were confirmed to be infected by *M. tuberculosis*.

**Table 3:**
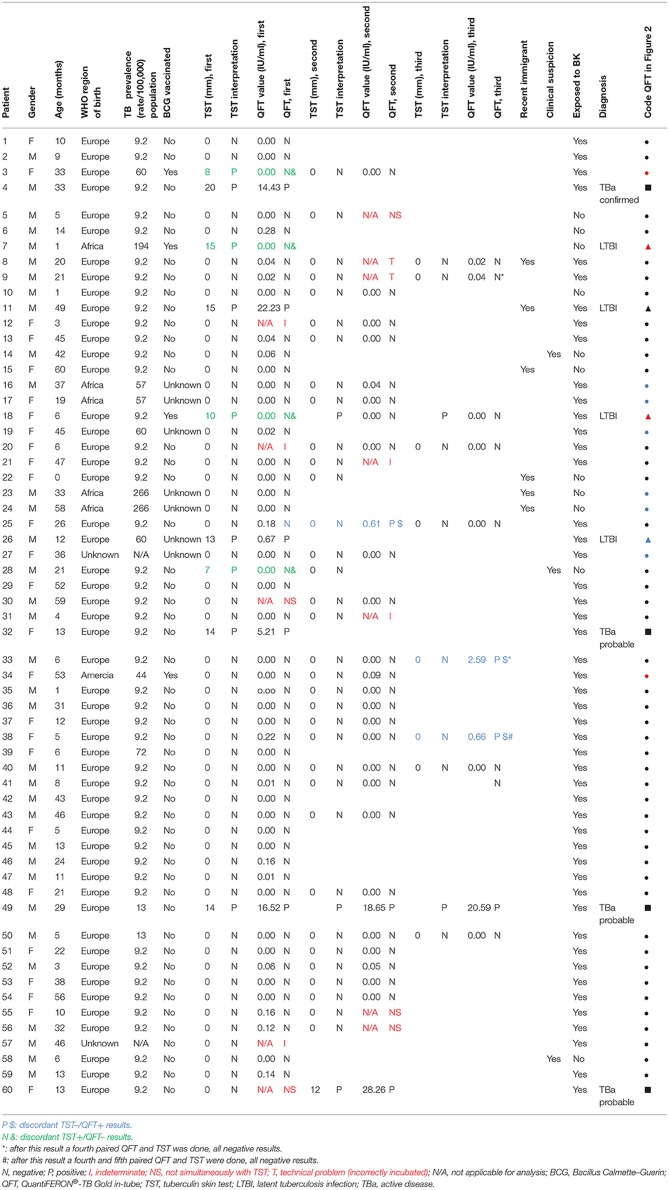
Results of all children (*n* = 60) with QFT/TST results.

Whereas, all children underwent TST at their initial evaluation, a paired and valid QFT result was available for 55/60 of them (92%). Three children had indeterminate QFT results and for two others QFT results were not obtained simultaneously with the TST. A second evaluation was obtained for 35/60 children (58%): for 33/35 children because they were recently exposed to TB but tested negative on the first TST and for 2/35 children for reasons related to clinical follow-up. At this second evaluation, two children had indeterminate QFT results, two had unevaluable samples for a pre-analytical reason (incorrectly incubated), three had samples not obtained simultaneously with the TST and two children were not QFT retested. As such, paired TST/QFT results were available for 26 of these 35 children. For the QFT analysis, a total of 109 blood samples were taken during the study from the 60 enrolled patients, of which 12 samples were excluded for the calculation of agreement.

The second round of TST testing was done, on average, 10 weeks after the first evaluation (between 4 and 33 weeks). Besides, among these 35 children, 8 had a third (TST/QFT) paired test, whereas another three infants (5, 6, and 21 months of age at the time of inclusion) had a fourth paired evaluation and one child (5 months old) even had a fifth controlled paired test. These supplemental blood tests have been asked for clinical purposes. Two children who presented with a TST ≥ 10 mm at the first evaluation were re-evaluated twice for QFT but without repetition of the TST.

The five children (ages 3, 4, 6, 46, and 47 months) with indeterminate QFT results following the first or second evaluation, had a negative TST and were considered as non-infected at the end of the follow-up period as they tested twice negative using the TST as the reference, and demonstrated at least two negative QFT. None of them had a known immunodeficiency. Reasons for indeterminate results included a Nil value > 8.00 IU/ml for two children (patients no. 31 and 57) and a PHA mitogen value minus Nil value of < 0.5 IU/ml for three children (patients no. 12, 20, and 21).

Among the 97 eligible samples for the test agreement analysis, the statistical analysis indicated a moderate agreement between both test results, when applying the cut-offs described in the methods section. The QFT results achieved a good classification for 88 samples (Kappa test 0.59, confidence interval 0.35–0.83). Taking the TST results as a reference, the positive predictive value of the QFT was 0.72, the negative predictive value was 0.93, the sensitivity of the QFT to identify *M. tuberculosis* infected children was 57%, and the specificity was 96% (Table 4). When we restricted the comparison of TST and QFT to the 79 samples from the 49 children who were not BCG vaccinated (Table 5), the degree of agreement became substantial (Kappa test 0.75, confidence interval 0.51−0.98) with 75 concordant samples (95%). Moreover, the negative predictive value of the QFT was 0.99.

**Table 4:** Comparison of tuberculin skin test and QuantiFERON® Gold in-tube test.

**QFT**	**TST**		
	**Positive**	**Negative**	**Total**
Positive	8	3	11
Negative	6	80	86
Total	14	83	97

*Six samples with discordant TST+IQFT– results from four children; three of them were BCG vaccinated children, one child had a TST value of 7 mm: exposure history to the assumed index case was not confirmed (smear-negative sputum examination and subsequently culture negative from the index case)*.

*Three samples with discordant TST–/QFT+ were from three children, their serial testing of TST and QFT negative at all other evaluations*.

*Number of observed agreement between QFT and TST: 88 samples (90.72% of the observations)*.

*Kappa test 0.59, 95% confidence interval 0.35–0.83*.

*QFT, QuantiFERON® Gold in-tube test; TST, tuberculin skin test*.

**Table 5:** Comparison of tuberculin skin test and QuantiFERON® Gold In-Tube test (without BCG).

**QFT**	**TST**		
	**Positive**	**Negative**	**Total**
Positive	7	3	10
Negative	1	68	69
Total	8	71	79

*Number of observed agreement between QFT and TST: 75 samples (94.94% of the observations)*.

*Kappa test 0.75, 95% confidence interval 0.52 to 0.98*.

*QFT, QuantiFERON® Gold In-Tube test; TST, Tuberculin Skin Test*.

Three children (patients no. 25, 33, and 38 in Table 3) who had been exposed and diagnosed as non-infected at the end of follow-up, had a positive QFT result whereas the simultaneous TST was negative (TST–/QFT+). Meanwhile, they received window prophylactic treatment until the second evaluation, 8 weeks after the latest exposure to infectious TB. Throughout the follow-up, subsequent QFT and TST controls for these children were all negative. For one 26-month-old child (patient no. 25), the first QFT performed at inclusion was negative, whereas the second result, 6 weeks later, revealed positive and the third, 1 year later, turned negative. Two other children (patients no. 33 and 38), respectively, 6 and 5 months old at inclusion, demonstrated their third QFT to be positive at, respectively, 9 and 14 months of age, while the two previous QFT results were negative and the TST stayed negative on the four testings. Extensive clinical follow-up of these three children for 2 years indicated that all of them remained in good health and were considered as not infected.

Four children (patients no. 3, 7, 18, and 28 in Table 3) had a total of six samples (6%) which were QFT negative, while simultaneous TST was positive (TST+/QFT–) (Table 4). Two of these children (patients no. 3 and 28) had a first TST induration of, respectively, 8 and 7 mm, whereas controls (TST and QFT) turned negative; both were finally considered as non-infected. As patient no. 3 had received a BCG vaccine, we regarded the first TST result as a false positive result. Patient no. 28, on the contrary, was not BCG vaccinated, moreover his exposure history to the assumed index case was not confirmed (smear-negative sputum examination and subsequently culture negative). As such his first TST result should also have been a false positive, we considered the initial positive TST in this child to be a non-specific skin reaction. The two other children (patients no. 7 and 18 in Table 3) came from a high TB burden country and had a record of BCG vaccination. We diagnosed LTBI based on their TST results.

Among the four children with LTBI, two had concordant positive paired (TST+/QFT+) test results, and two had discordant TST+/QFT– test results. Among the children with a final diagnosis of pulmonary TB, all had concordant positive (TST+/QFT+) test results on the six collected paired samples.

**Figure 2:**
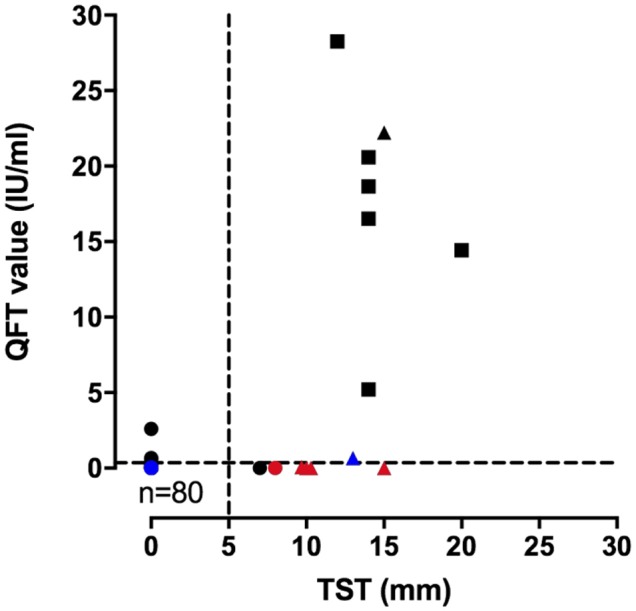
Correlation of QuantiFERON interferon-γ values (IU/ml) and TST diameter of induration (mm). The results of the QuantiFERON interferon-γ values and the TST diameter of induration were compared for 97 samples including 52 children diagnosed as not infected (•), but 85 samples; five children with a diagnosis of latent tuberculosis infection (▴), but six samples; four children with a diagnosis of pulmonary TB (◾), but six samples. BCG-vaccinated children were identified with a red color, BCG-unvaccinated children with a black color, and unknown BCG status children with a blue color. Each dot corresponds to a single sample. The value of the r Spearman correlation index is 0.36 (95% confidence interval 0.17–0.53).

To investigate the association between the TST and the QFT result, we also compared IFN-γ concentrations or the QFT value (IU/ml) (TB Antigen minus Nil) to the induration size of the TST (mm) for 97 blood samples (Figure 2). A poor correlation between both results was obtained, as shown by the Spearman's rank correlation coefficient (0.36, 95% confidence interval 0.17–0.53). However, we observed the highest QFT values in children with the largest diameter of TST induration.

## Discussion

In our study, we evaluated the accuracy of QFT as a screening test in young children at risk for TB infection, in a low TB prevalence country. In the absence of a gold standard to diagnose LTBI, we compared QFT with TST results. Considering TST as the reference, we observed a moderate agreement between QFT and TST (90.72%, Kappa 0.59) with 9% of paired (TST/QFT) tests being discordant. However, in children with no previous BCG vaccination, there was a good agreement (95%, Kappa 0.75).

Comparative studies in low TB burden countries demonstrated lower levels of agreement than observed in our study. Lighter et al. found an overall agreement of 55% (Kappa 0.17) in children with LTBI, which increased to 72% (Kappa 0.31) considering children who had not been BCG-vaccinated. Agreement was stronger (91%), in the subgroup of children not vaccinated and presenting a TST > 15 mm (18).

Spicer et al. evaluated the correspondence between TST and the T-SPOT.TB test, another IGRA, among 107 young adopted children. Overall agreement was 80.8% (Kappa 0.68); however, BCG vaccination reports were available only in 72% of the cohort and an unknown higher BCG coverage in these adoptees could have influenced the agreement (19).

The accuracy and reliability of IGRAs among young children are not yet well-defined. Limitations of this test includes, in comparison to TST: lack of data in very young children, increased frequency of indeterminate results in children <5 years old (9) and the inconveniences of doing phlebotomy in small children: at least two nurses are required and obtaining a minimum blood volume of 4 ml is not always easy in young children.

Indeterminate QFT results are (9, 20) observed in pediatric populations in 0–35% (18, 21, 22). A high rate of indeterminate results is described in immune deficient patients, non-TB related acute infections or active severe TB disease (23). In our cohort of young children, the rate of indeterminate QFT, following the manufacturer's recommendations, was 4.5%. After follow-up, we finally considered these five children as not infected. Our results suggest that age *per se* is not a factor increasing the rate of indeterminate results.

In this study, four children presented TST+/QFT– discordant results. For two of them, this was most likely explained by a false positive TST as the TST turned negative on subsequent testing. For the two other children, we could not determine if the TST was false-positive or the QFT false-negative. Both were BCG vaccinated and came from a high-burden country.

Most experts consider TST+/IGRA– discordant results as false positive TST results in recent BCG-vaccinated individuals (24–26). Seddon and colleagues evaluated the impact of BCG vaccination on TST results and observed that the BCG vaccination affected the TST response in IGRA-negative children aged <5 years, but this effect waned rapidly with age (26). However, in young at-risk children, these TST+/IGRA– could be due to the low capacity of young children to produce IFN-γ and other cytokines in response to mycobacterial antigens (27–30). To overcome underdiagnosis in BCG-vaccinated young children tested for LTBI, Pavic recommends using both tests and considering the child infected if either one or both are positive (25).

Reverse discordant results (3%), TST–/QFT+, were observed in three children, exposed to a contagious TB case. They presented, each on one occasion, a positive quantitative QFT value that ranged from 0.61 to 2.59. Finally, we considered these children as not infected at the end of the follow-up period. The positive QFT results were preceded and followed by a negative QFT result, and the TST was three times negative for all three children. Reversion of positive QFT results to negative results have been described previously and may result from transient immune responses in children exposed to *M. Tuberculosis* (31). They have been reported to be more frequent for quantitative QFT values ranging from 0.35 to 4.00 IU/ml (29, 32).

In this study, the positivity threshold recommended by the manufacturer, i.e., 0.35 IU/ml, was applied for the QFT value (TB Antigen minus Nil). However, some discussion had arisen about the need to adjust the positivity cut-off for the QFT value. Lombardi and colleagues showed that children <5 years old with LTBI produced lower levels of INF-γ in response to TB antigens (median 1.96 IU/ml) than those with active TB (median 10 IU/ml) (29). Similarly, Andrews et al. concluded from the results of serial QFT testing in young children in a large study in South Africa, that QFT conversion with values higher than 4.00 IU/ml was associated with a substantially increased risk (28%) of developing TB disease (32). If we had considered a threshold of more than 4.00 IU/ml, we wouldn't have observed any discordant TST–/QFT+ results. On the one hand, three children in our cohort who presented TST–/QFT+ results could have been diagnosed as LTBI considering the QFT-positive testing, following Pavics recommendations (25). The QFT cut-off of 0.35 IU/ml turned these cases positive (QFT values of 0.61, 0.66, and 2.59) while they would have been negative using a threshold of more than 4.00 IU/ml. On the other hand, one child (patient no. 26 in Table 3), 12 months old, originating from Eastern Europe, without any documented BCG status, and exposed to a contagious pulmonary TB case, was considered as an LTBI, based on a positive TST. His QFT test demonstrated a “positive” quantitative value of 0.69 IU/ml, slightly above the limit but not as high as other children diagnosed as LTBI or children with TB disease. If a threshold of >4.00 IU/ml would have been used, this would give a discordant TST+/QFT– result.

Among other children labeled as LTBI, patient no. 11 also requires special consideration. Its quantitative QFT value was as high as those observed in active TB children and far above the median value presented by Lombardi for LTBI (29). But only one test was available in this case, during clinical follow-up there was no suspicion of development of active TB disease. The distinction between infection and subclinical disease or imminent progression to TB disease may be blurred. Results of serial testing turned out to be very informative. Extended follow-up of children with discordant (TST–/QFT+) results had low predictive value for TB and were associated with high rates of reversion (32). In our study, we concluded based on follow-up testing that these QFT results were most likely false positive results.

A limitation of this study is that we evaluated the third generation QFT which differs from the actual fourth generation of the IGRA QuantiFERON-TB Gold Plus that uses two antigen tubes (TB1 and TB2) instead of only one. The previous single tube QFT contained peptides from three different antigens, ESAT-6, CFP-10, and TB7.7. The two tubes of the new version only contain peptides from ESAT-6 and CF-10 antigens, but shorter peptides were added with the hope to detect CD8+ T cell responses (in TB2 tube) in addition to CD4+ T cell responses (in TB1 tube). This new version has been marketed with the prediction that it is more sensitive for the immunocompromised patient. Literature shows an overall good agreement between both tests, no prospective studies on young children have been published yet (33, 34).

## Conclusion

In this prospective study of a cohort of children aged <5 years, at high risk of TB in a low TB prevalence country, QFT, one of the commercially available IGRA tests, proved to have substantial agreement with TST. Our study population and observations made us aware of false positive and false negative QFT results. In this young age group, recent BCG vaccination seemed to influence TST results and was more likely associated with false positive TST than with false negative QFT.

Indeterminate results were observed, but not in high percentages in otherwise healthy children. Further studies should help with strengthening our observations, particularly to evaluate discordant results and the threshold for the QTF value (TB Antigen minus Nil) to avoid false positive QFT results.

Currently, in the very young population (<1 year old), we advise to evaluate both TST and IGRA after the window period of 8 weeks and to continue treatment if one or either test is positive.

## Ethics Statement

The study was approved by the Ethics Committee of the St. Pierre University Hospital, reference AK/09-05-27/3746AD.

## Author Contributions

SD, FMa, and FMo conceived the idea and developed key concepts in the manuscript, contributed to subsequent revisions of the manuscript. SD wrote the first draft of the manuscript. SD, VC, and JL collected and analyzed data, contributed to the first draft and revisions of the manuscript. SD and VC performed the statistical analysis. PS and KV contributed to subsequent revisions of the manuscript. All authors contributed to manuscript revision, read and approved the submitted version.

### Conflict of Interest Statement

The authors declare that the research was conducted in the absence of any commercial or financial relationships that could be construed as a potential conflict of interest.

## Abbreviations

BCG: Bacillus Calmette–Guérin
IGRA: Interferon gamma release assay
IFN-γ: interferon-gamma
IU/ml: International Units/milliliter
LTBI: Latent tuberculosis infection
*M*.: *Mycobacterium*
mm: millimeter
NTM: Non-Tuberculous Mycobacteria
number: no
QFT: QuantiFERON®-TB Gold In-Tube test
TB: tuberculosis
TST: tuberculin skin test
WHO: World Health Organization.
